# The impact of different preanalytical methods related to CA 15-3 determination in frozen human blood samples: a systematic review

**DOI:** 10.1186/s13643-021-01631-7

**Published:** 2021-04-09

**Authors:** Luigi Coppola, Alessandra Cianflone, Katia Pane, Monica Franzese, Peppino Mirabelli, Marco Salvatore

**Affiliations:** IRCCS SDN, Via E. Gianturco, 80143 Naples, Italy

**Keywords:** Frozen human blood, CA 15-3 determination, Preanalytical phase, Tumor biomarkers, Biochemical indicators

## Abstract

**Background:**

The determination of CA 15-3 is useful for monitoring breast cancer patients. Several retrospective studies determined CA 15-3 levels in frozen samples to evaluate the sensitivity and specificity of novel biomarkers in relation to breast cancer; however, freeze-thaw cycles, as well as preanalytical variables before sample storage, are not always reported. Here, we analyzed the current scientific literature to identify possible critical aspects related to CA 15-3 determination in frozen-stored human serum/plasma samples.

**Methods:**

We obtained data from 4 different bibliographic databases: Web of Science, Embase, PubMed, and Cochrane Library. We followed the PRISMA guidelines to screen and select the eligible articles discussed in the final revision.

**Results:**

Initially, 674 scientific papers were evaluated, and after the application of the screening and eligibility criteria, 18 studies were included in the qualitative synthesis. The analysis reported an important level of heterogeneity concerning the preanalytical phase before sample storage.

**Conclusion:**

Although advances in healthcare have been achieved using certified workflows in medical diagnostics, standardized preanalytical processes are not always applied when referring to frozen-stored biosamples. Biobanks will guarantee the best possible conditions for the storage of human biological samples to be used in clinical research. The use of certified bioresources will favor the optimal development and introduction of new disease biomarkers.

**Supplementary Information:**

The online version contains supplementary material available at 10.1186/s13643-021-01631-7.

## Background

Biomarkers are biomolecules that usually serve as indicators of pathological processes or for having clinical information about pharmacological responses to drug treatment [1]. Due to their clinical usefulness, biomarker discovery and validation represent one of the pillar strategies for research in the field of personalized medicine [2] for better diagnosis and prognosis [3, 4]. However, it is important to consider that the life-cycle stages of biosamples, i.e., collection, accession, acquisition, identification, preservation, long-term storage, quality control (QC), transport, and sharing of biomaterials, represent a major source of heterogeneity among biorepositories. Consequently, the use of frozen biosamples, without the application of standardized storage procedures, can negatively affect biomarker identification and validation phases [5] as well as result comparisons across studies.

In this context, to better understand the critical aspects related to the management of human biological samples, we decided to evaluate how the preanalytical variables and storage conditions (including freeze-thaw cycles) are correctly reported in the literature for CA 15-3, a commonly used biomarker for monitoring breast cancer (BC). We selected this marker since it is widely used in clinical practice for patient stratification in retrospective case-control studies as well as for performance comparison with novel introduced biomarkers. In doing so, we reviewed the literature of the past 10 years on different scientific databases (PubMed, Embase, Web of Science, and Cochrane Library), focusing our attention on technical approaches (frozen or fresh samples, storage temperature, type of biological samples used, cutoff adopted, etc.) and selecting the CA 15-3 determinations obtained from thawed samples. Generally, CA 15-3 represents the soluble form of mucin-1 (MUC-1) antigen. This surface protein is upregulated (10-fold higher than in adjacent normal glandular epithelium) on the surface of breast cancer cells [6], and after being shed from the BC cell surface, it is released into the bloodstream and used as a disease biomarker, as stated by different scientific studies [7, 8]. Despite its association with BC cells, an increase in CA 15-3 can be detected in some benign conditions, such as liver disease and benign breast, lung, or ovarian disease [9]; therefore, it is not considered a specific BC biomarker for diagnostic purposes.

However, the European Group on Tumor Markers (EGTM), in agreement with the National Academy of Clinical Biochemistry (NACB) guidelines, suggests that increasing levels of serum tumor markers may often precede disease recurrence [10, 11]. Thus, CA 15-3 levels, according to the EGTM in agreement with the NACB guidelines, serve mainly for monitoring BC patients at risk of developing metastatic disease since increasing levels of CA 15-3 may often precede disease recurrence [10, 11].

Technically, the CA 15-3 value is determined in fresh blood serum samples; however, in biomedical research, the determination of biomarkers from thawed human biological samples is frequently carried out to evaluate a new analytical technique or for comparison with other biomarkers. In this regard, several studies systematically investigated the effect of storage conditions on blood samples for the measurement of different analytes [12–14]. While CA 15-3 preanalytical variables have already been studied in previous works [15–17], the preanalytical factors influencing CA 15-3 determination in frozen human blood samples have never been studied in detail. For this reason, we performed a critical review of literature studies on CA 15-3 determination in frozen-thawed samples. Here, we emphasize study characteristics based on preanalytical variables and discuss the results in light of standard procedures for the proper biobanking and handling of human biosamples [5]. We believe that correct storage of biological samples is necessary not only for retrospective CA 15-3 determination but also for the identification and validation of novel clinical biomarkers. In this way, scientific results will be more comparable across studies, especially for the introduction of novel biomarkers.

## Materials and methods

The paper was prepared based on the Preferred Reporting Items for Systematic Reviews and Meta-Analyses (PRISMA) standards and guidelines (Additional file 1) [18]. All data originate from previously published experiments in international peer-reviewed journals.

### Search strategy and eligibility criteria

For eligibility criteria on study characteristics, we included English peer-reviewed papers involving CA 15-3 in human fluids such as serum, plasma, or whole blood and excluded in vitro studies. Only original articles with publication status information within a time interval of 10 years (January 2010 up to April 2020) were included, while case reports, reviews, and editorials were excluded. All studies were identified by searching the PubMed, Embase, Web of Science, and Cochrane Library databases by using the following search keywords: “ca 15-3,” “ca 15.3,” “muc1,” “mucin 1,” “carbohydrate antigen 15–3,” “carbohydrate antigen 15.3,” “serum,” “plasma,” and “blood” with the last search date on 1 April 2020.

### Data extraction and collection

Two authors carried out article searches and data collection independently (L.C. and A.C.). A third reviewer (K.P.) independently carried out data extraction and reviewed the selected published articles to confirm that they met the inclusion criteria. The extraction of data for the following predetermined variables was performed: study design, study period, the outcome of the study, and the presence of follow-up in time-to-event studies, i.e., follow-up length, age, patient subgroups, age, and sex-matched case-control, association with clinical-pathological features, statistical methods for CA 15-3 determination, and biospecimen source as described below in the data item section. Any disagreements that arose between the reviewers were resolved through discussion with a fourth, fifth, or sixth reviewer (P.M., M.F., and M.S.).

### Study selection

**Fig. 1:**
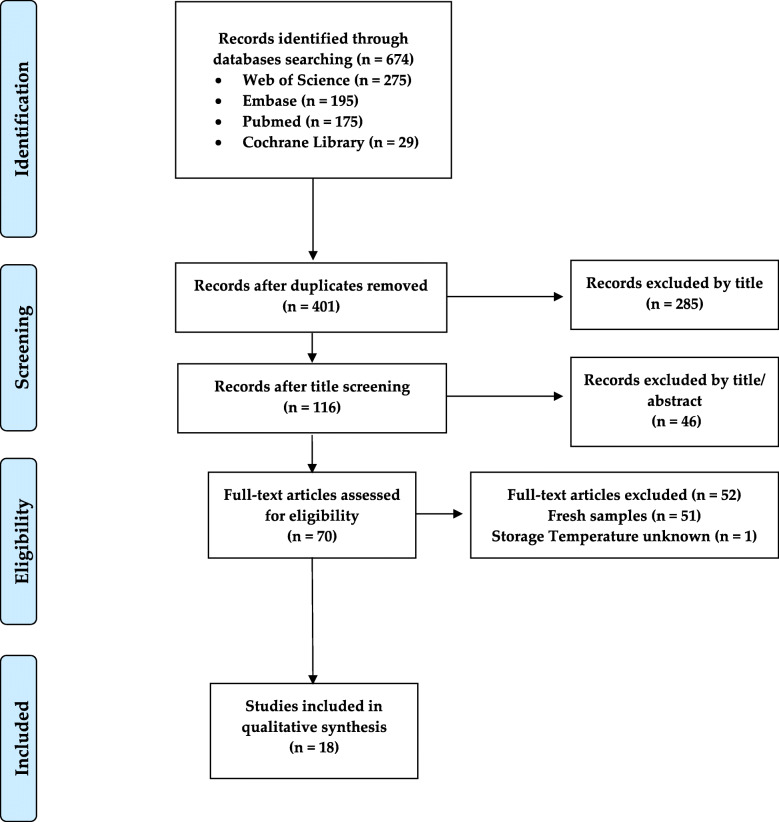
PRISMA flow diagram: flow diagram of the identification, screening, and inclusion of the 18 eligible studies according to the PRISMA statement

Circulating tumor biomarker detection has clinical utility for patient management and is determined using fresh serum or plasma samples [19]. Conversely, frozen samples are required for retrospective analysis focused on the identification or validation of novel biomarkers as well as the establishment of new analytical technologies. For this systematic review, we included studies on frozen-thawed samples according to the PRISMA flow diagram shown in Fig. 1. Using four different databases (PubMed, Embase, Web of Science, and Cochrane Library), we selected papers with retrospective, prospective, or cross-sectional studies, resulting in a total of 674 papers. Initial screening of the titles led to the exclusion of 285 articles, while title and abstract screening led to the exclusion of 46 articles. Among the 70 full-text articles assessed for eligibility, we excluded 52 full-text articles since the CA 15-3 determinations were not carried out on thawed human biological samples, and we excluded 1 full-text article because the sample storage temperature was unknown [20]. Finally, we included 18 full-text articles for the final review; Excel (Microsoft Office 2019, software) was used to remove duplicate articles.

### Data items

Data items for searching papers were specifically related to the presence of the following variables of interest: biospecimen type, i.e., human sample type, fresh or frozen human samples, and sample preanalytical variables, such as (i) the handling of aliquots of the total biospecimen; (ii) freeze-thaw cycle and temperature; (iii) aliquot centrifugation before testing; and (iv) interim aliquot storage until analysis. Searching for CA 15-3 determination on frozen-thawed samples, we assumed that fresh samples could also be included in the selected papers since both sample types can be assayed concomitantly. Since CA 15-3 is not a specific BC biomarker, we simplified the disease and/or clinical outcome search to cover more literature findings. Thus, different diseases and outcomes (disease monitoring, recurrence, or follow-up) are included in the final selected studies.

### Summary measures

We assumed as principal summary measure the CA 15-3 cutoff value as the difference in mean concentrations between the case groups and the control groups with the standard mean error or standard deviation for statistical significance as reported in each study.

### Risk of bias

We carried out the risk of bias assessment by objectively evaluating the selected publications with the following questions: (1) Can we find confounding factors for sample handling? (2) Can we reproduce the sample freeze-thaw cycle? (3) Are we confident in the appropriate balance of the sample sizes of the case and control groups? We assessed the risk of bias across studies by using the Newcastle-Ottawa Scale (NOS) for case-control study quality assessment [21].

### Quality assessment of selected studies

We carried out a quality assessment using the Newcastle-Ottawa Scale (NOS) for the case-control studies (Additional file 2) [21]. We judged study group comparability items (maximum two stars for a single item) based on arbitrarily important factors, such as one star to a study that explained the freeze-thaw cycle, such as (i) the handling of aliquots of the total biospecimen; (ii) freeze-thaw cycles and temperatures; (iii) aliquot centrifugation before testing; and (iv) interim aliquot storage until analysis, and one star to a study that had a balanced case-control study sample size. The sample size balance estimation is arbitrarily defined using the threshold of *n* ≤ 10 samples for group comparison.

## Results

### Study characteristics

The final included articles (*n* = 18) encompassed case-control studies based on 17 retrospective study designs [22–38] and one prospective study [39]. The sample size across studies ranged from a minimum of 30 [39] to a maximum of 472 [27]. Table 1 reports a summary of the findings of the 18 papers selected for the critical review classified according to study type, the number of studies, biospecimen type, storage temperature (°C), patient status/disease, measurement unit, and the kit or instrument used. Specifically, according to the biospecimen type used for CA 15-3 determination in the 18 included papers, 14 studies (approximately 80%) used serum samples [22–32, 34, 35, 39], while the remaining 4 were structured as follows: (i) Zajkowska et al. used exclusively plasma samples [33], (ii) Christenson et al. focused on methodological comparison and assayed CA 15-3 in both serum and plasma samples [36], (iii) Saba et al. measured CA 15-3 in both serum and pleural effusion [37]; and (iv) Laidi et al. determined CA 15-3 in serum and saliva biosamples [38]. Regarding the preanalytical variable of storage temperature, 9 studies out of the 18 (50%) [26–30, 32, 34, 35] stored biosamples at − 80 °C until analysis, 6 studies stored biosamples at − 20 °C [22–25, 37, 39], one study [31] stored biosamples at − 70 °C, one study stored plasma at − 85 °C [33], and only Christenson et al. assayed CA 15-3 from biosamples stored at both − 20 °C and −70 °C [36].

**Table 1:** Summary of the findings of the 18 eligible articles on CA 15-3 determination from thawed human biosamples

	No. of studies [reference]
**Study type**	
Retrospective study	17 [22–38]
Prospective study	1 [39]
**Biospecimen type**	
Serum	14 [22–32, 34, 35, 39]
Plasma	1 [33]
Serum and plasma	1 [36]
Serum and pleural effusion	1[37]
Serum and saliva	1 [38]
**Storage temperature (°C)**	
− 80	9 [26–30, 32, 34, 35, 38]
− 20	6 [22–25, 37, 39]
− 70	1 [31]
− 85	1 [33]
− 20; − 70	1 [36]
**Patient status/disease**	
Breast cancer	14 [22–29, 31–35, 38]
Pleural effusion	1 [37]
Adnexal masses and ovarian cancer	1 [30]
Pregnancy	1 [39]
Multiple disease	1 [36]
**Measurement unit**	
U/mL	15 [22–30, 32–34, 36, 38, 39]
ng/mL	2 [35, 37]
U/L	1 [31]
**Kit or instrument**	
CLEIA	4 [27, 28, 34, 35]
EIA	3 [25, 29, 39]
ELISA	3 [22, 37, 38]
HumaReader Plus made by HUMAN GmbH	2 [23, 24]
MEIA	2 [30, 31]
RIA	2 [26, 32]
CMIA	1 [33])
Vista vs Advia Centaur	1 [36]

In the selected papers, the CA 15-3 measurement was mainly (approximately 80%) evaluated for monitoring BC; in fact, 14 out of the 18 articles were focused on this pathology [22–29, 31–35, 38]. Interestingly, 4 articles underlined that CA 15-3 determination is also executed for other needs; indeed, this tumor marker was even analyzed in adnexal mass patients and in patients with pleural effusion [30, 37]. Additionally, the accuracy of CA 15-3 determination was also evaluated in 30 pregnant women, and in one case, it was evaluated in an instrument comparison study for measuring multiple disease biomarkers from BC patient samples [36, 39].

We also studied other variables, such as information related to the kits or instruments used and cutoff values. In 15 of the 18 studies, the measurement unit for CA 15-3 was the difference in CA 15-3 mean concentration between the case groups and the control groups expressed in U/mL with the standard mean error or standard deviation for statistical significance; in Saba et al. [37] and Tang et al. [35], ng/mL was reported; and in Metwally et al. [31], U/L was reported. To determine CA 15-3, 4 selected studies used chemiluminescent enzyme immunoassays (CLEIAs) [27, 28, 34, 35]; 3 studies used enzyme-linked immunosorbent assays (ELISAs) [22, 37, 38]; and in other studies, enzyme immunoassays (EIAs) [25, 29, 39], microparticle enzyme immunoassays (MEIAs) [30, 31], radioimmunoassays (RIAs) [26, 32], and chemiluminescent microparticle immunoassays (CMIAs) [33] were used. Finally, 3 out of the 18 papers reported the semiautomated or automated instrument used [23, 24, 36]. Additionally, it is known that interim storage at low temperatures can affect the quality of human biological samples [40, 41]; unfortunately, only 1 (36) out of the 18 selected papers reported the freezing period before CA 15-3 determination.

### Synthesis of results

Overall, the data in Table 1 emphasize that CA 15-3 determination is heterogeneous and is performed through different technical approaches. Critical differences were found in relation to the storage temperature, measurement unit, and kit or instrument used for testing new possible approaches for translational research. Additionally, it is necessary to emphasize other missing information, such as (i) the hemolysis of biological samples, (ii) the frozen storage duration from all papers except that of Christenson et al. [36], and (iii) how frozen samples were thawed before CA 15-3 determination. A more detailed description of the characteristics of the studies within the 18 selected articles is reported in Table 2.

**Table 2:** Synthesis of study characteristics

Study/year	Case/control	Study period	Outcomes	Methods	Age (years)	Subgroups	Age-/sex-matched case-control	Follow-up length	Clinical-pathological association/correlation	CA 15-3 cutoff (U/mL)	Kit or instrument	Sample type	Store temperature (°C)
Chukwurah et al. 2018 [22]	(89/21) naive BC vs healthy	2015–2017	CA 15-3 for BC surveillance	Mean ± SD	Age range (29–65)	Yes	Yes	Up to 3 and 6 months	Yes	< 35	ELISA	Serum	− 20
Mahmood et al. 2016 [23]	(70/20) premenopausal BC vs healthy	–	Ferritin vs CA 15-3 in BC	Mean ± SD; ANOVA	Age range (20–50)	Yes	Yes	–	Yes	< 119.39	HumaReader Plus made by HUMAN GmbH	Serum	− 20
Said 2019 [24]	(84/71) BC vs healthy	2016–2017	CBC with ALP and LDH for BC prognosis	ROC analysis	Mean age (32.7 ± 18.3)	No	Yes	–	No	< 15.3	HumaReader Plus made by HUMAN GmbH	Serum	− 20
Moazzezy et al. 2014 [25]	(30/30) BC naive vs healthy	2012–2013	Preoperative and clinicopathological BC features	Mean ± SEM unpaired Student’s *t*-test; regression analysis	Mean age 48 (23–87)	Yes	Yes	–	Yes	< 30	EIA	Serum	− 20
El-Moneim Ebied et al. 2013 [26]	(50/50) BC naive vs healthy	2010–2010	Diagnostic, prognostic, and follow-up value of CA 15-3 in BC	Mean ± SE. ROC analysis; univariate survival analysis KM	Mean age (41.73 ± 12.2)	Yes	No (comparable age (40.18 ± 11.05 years))	2 years	Yes	< 25	RIA	Serum	− 80
Svobodova et al. 2018 [27]	(412/60) BC-DR vs NDR	–	TPS, CEA, and CA 15-3 (months 1–3–6 after surgery). DR was recorded between 7 and 12 months after surgery	Mean ± SD and Wilcoxon test	Median age 61	No	Yes	1, 3, and 6 months (after surgery). 7 and 12 months for DR	No	< 30	CLEIA	Serum	− 80
Pedersen et al. 2013 [28]	(83/24) BC metastatic vs LR	2004– 2010	CA 15-3, CEA, and HER2 in the early diagnosis of metastatic HER2+ BC	95% confidence intervals	Median age 59.1 (30–86)	Yes	–	–	Yes	< 32.4	CLEIA	Serum	− 80
Di Gioia et al. 2015 [29]	(241) BC patients	1998–2007	Prognostic value of HER2 and CA 15-3 and DFS and cancer-specific survival in preoperative BC	Wilcoxon rank-sum test	Median age 57 (range 29–89)	Yes	–	7–91.4 months	Yes	< 24	EIA	Serum	− 80
Sen et al. 2011 [30]	(13/12/25/19) OV stage I/II, OV stage III/IV; benign ovarian vs healthy	2009–2011	CA 15-3, IL-6, Leptin, VEGF, and CRP for ovarian cancer prediction	Mean difference and Mann-Whitney *U*-test; Wilcoxon; Kruskal-Wallis test	OV stage I/II (44.6±4.65), OV stage III/IV (49.6±6.4); benign ovarian (41.6±10.3); HC (40.1±8.9)	No	No	–	Yes	< 30	MEIA	Serum	− 80
Metwally et al. 2010 [31]	(44/15) BC patients vs healthy	2009–2010	Diagnostic value of VEGF, IL-18, and NO levels in BC patients	Mean and SEM difference; ROC curve	Median age 36 (23–56)	Yes	–	–	Yes	< 11.2	MEIA	Serum	− 70
Hewala et al. 2012 [32]	(35/35) BC naive vs healthy	01/2010–08/2010	sFas and p53 protein vs CA 15-3 to monitor BC patients	Mean ± SE; Kruskal-Wallis test; ROC analysis	Case mean age (43.73 ± 12.2); HC mean age (42.18 ± 11.05)	Yes	Yes (comparable mean age)	1 year	Yes	< 23	RIA	Serum	− 80
Zajkowska et al. 2020 [33]	(120/28/32) BC patients/benign tumors (adenoma 10), fibroadenoma (18) vs healthy	–	VEGF plasma concentrations alone or combination with CA 15-3 in BC patients	Median range; ROC curve	BC cases median age 58 years, range (39–83); benign tumors 48, range (36–71)	Yes	Yes	–	Yes	< 18.45	CMIA	Plasma	− 85
Zaleski et al. 2018 [34]	(55/20/28) BC patients/benign breast disease vs healthy	2010–2013	miRNAs compared with CA 15-3 in BC monitoring	Mean; ROC curve	BC cases, 44 median age (20.1–64.5); benign breast disease, 54 median age (24.2–81.8); HC, 59 median age (32.6–85.8)	Yes	Yes	NA	Yes	< 27	CLEIA	Serum	− 80
Tang et al. 2018 [35]	(40/40/40) BC patients/benign mammary hyperplasia vs healthy	2004–2005	ALU115, ALU247/115, CEA, and CA15 in BC	Median and Mann-Whitney *U*-test	Median age 48 years, range 28–64. BC cases median age 46, HC median age 45	Yes	Yes	NA	Yes	< 31.3	CLEIA	Serum	− 80
Christenson et al. 2011 [36]	(66/77) CA 15-3 serum vs plasma	–	Validation of CA 15-3, CA 19-9, CA 125 II, CEA, and AFP for instrument comparison	Mean; ROC curve	All patient samples > 18	No	–	Day 0 and 1 year	No	< 42.1	Vista vs Advia Centaur	Serum and plasma	− 20; − 70
Saba et al. 2017 [37]	(44/49) malignant pleural fluid vs benign pleural fluid	2013–2014	CA 15-3 and NSE solely or in pleural fluid	ROC curve	Malignant cases mean age 66.6 ± 16.6 years versus benign pleural effusion controls 73.4 ± 16.5	No	No	NA	–	< 13.9 and < 6.68 L*	ELISA	Serum and pleural effusion	− 20
Laidi et al. 2014 [38]	(29/31) BC patients vs healthy	–	Correlation of salivary and serum CA 15-3 in BC patients	Median value; Mann-Whitney test	Mean age cases 7.24 ± 9.52 and HCs 43.45 ± 14.72	No	No	NA	Yes (lymph node status)	< 16.7	ELISA	Serum and saliva	− 80
Ercan et al. 2012 [39]	30 healthy pregnant women	–	Specificity of CA-125, CA 15-3, CA 19-9, and CEA during pregnancy	ANOVAs	Median age 29 years range 28–64	No	No	10–36 weeks	No	<25	EIA	Serum	− 20

*BC* breast cancer, *DFS* disease-free survival, *DR* disease recurrence, *LR* local recurrence, *NA* not accessible

Note: *Different measurement unit for CA 15-3 cutoff value

### Risk of bias across studies

We assessed the risk of bias across studies by evaluating the bias in the selection of cases and controls, as well as the comparability across studies for important factors such as the freeze-thaw cycle and handling to allow the reproducibility of the research and expose bias (Additional file 2). Cases in most studies were based on self-medical reports and/or hospital examinations without multiple independent medical validations. Indeed, the consensus diagnosis performed by three specialists is described only in the paper by Sen et al. on ovarian cancer and adnexal mass investigation [30]. Most case-control studies are based on consecutive sampling from hospitals or universities/hospitals. This is an important bias that is generally present in the majority of the studies based on hospital-community controls rather than those based on population-based controls. In this context, confounding factors may arise because most of the “healthy subject” controls are referred to as “healthy volunteers” without further description. Indeed, 6 out of 18 articles clearly stated that controls had no history of the disease (endpoint), and only Said et al. described a full history of the interview for all cases and controls [24]. A risk of bias across the studies within all eligible articles was found when comparing the freeze-thaw cycle and handling of samples. Among the 18 eligible articles, that of Christenson et al. [36] described most of the preanalytical variables considered in this systematic review. Indeed, they reported sera and plasma handling, storage stability at different temperatures (Table 1), the origin of fresh-frozen samples from the commercial blood bank, and sample thawing for CA 15-3 determination at day 0 and after 1 year of storage.

Heterogeneity in the case ascertainment of exposure (no surgical record linkage) was found. Additionally, the case-control nonresponse rate was described in only one study [36]. Several studies focused on CA 15-3 determination for BC monitoring, such as disease recurrence or treatment follow-up. We found that Pedersen et al. [28] studied the risk of bias in sample handling since serum HER2 determination was carried out prospectively (on fresh samples), while serum CEA and CA 15-3 were analyzed retrospectively in samples stored at − 80 °C. Additionally, the period of case follow-up was not reported in detail. Furthermore, Chukwurah et al. carried out BC surveillance in Nigerian women. Their CA 15-3 levels may be difficult to compare to those of other studies during the follow-up period (3 to 6 months, probably due to the Nigerian program’s screening policy) [22]. Additionally, a risk of bias was present in the Svobodova et al. follow-up case-control study, whose case monitoring follow-up after surgery was carried out in different centers for longer intervals [27].

We found selection bias in some eligible studies that did not explain whether eligible patients were enrolled consecutively or randomly (Additional file 2).

## Discussion

The present work aimed to evaluate the impact of preanalytical conditions related to CA 15-3 determination in frozen human samples and to highlight the importance of using standardized procedures across studies for comparing results across studies. The selected papers encompass 18 case-control studies focused on CA 15-3 determination in frozen serum or plasma samples. Most of these studies aimed to evaluate the sensitivity and specificity of novel circulating biomarkers in comparison to CA 15-3 in relation to BC or additional diagnostic applications of CA 15-3 apart from BC. The bibliometric analysis performed in this study revealed that several technical data related to the processing, storage period, and thawing conditions were missing or only poorly reported. Overall, the data in Table 1 emphasize that CA 15-3 determination was heterogeneous and was performed through different technical approaches. Critical differences were found in relationship to the storage temperature, measurement unit, and kit or instrument used for testing new possible approaches for translational research. Additionally, it is necessary to emphasize other missing information, such as (i) the hemolysis of biological samples, (ii) the frozen storage duration from all papers except that of Christenson et al. [36], and (iii) how frozen samples were thawed before CA 15-3 determination. These variables led to heterogeneous experimental conditions for CA 15-3 determination in the thawed samples across the included studies. Among the 18 eligible articles, only Christenson et al. [36] described that frozen samples (− 70 °C) were tested within 1 year from the date of collection, and samples were thawed at room temperature for 30 min and recentrifuged at 1500×*g* for 2–5 min [36]. Although such reporting might seem “ancillary,” it is important to adopt common collection, processing, storage, and thawing procedures for the management of biological samples for data reproducibility within the international scientific community. In this context, samples from biobanks might also guarantee higher-quality biosamples [42, 43] by minimizing the effect of preanalytical variables on the sample life cycle, including storage for a long period. One study out of the 18 selected papers assayed CA 15-3 in biological samples obtained from a biobank, i.e., Zaleski et al. from the Biofluid Biobank of the University Hospital Bonn [34]. In this case, it is important to highlight that obtaining samples from a biobank is optimal. Indeed, a biobank processes its samples following a standardized procedure [44]. Moreover, the same sample can be used by different researchers to produce comparable results and validate different markers in the same patient cohort.

Regarding the limitations of our study, we focused on 10 years (January 2010–April 2020) of literature studies for CA 15-3 determination and preanalytical variables affecting frozen biosample handling to evaluate a manageable number of articles and the most recent articles; however, we are aware that preanalytical variables affect not only frozen human samples but also likely every type of biosample.

## Conclusions

One frontier of precision medicine is to identify noninvasive novel tumor biomarkers; consequently, the proper use of frozen-stored biosamples is of utmost importance. Here, we highlighted that heterogeneous preanalytical variables and storage conditions are applied when retrospective case-control studies are performed. This highlights an important source of variability across studies, which leads to results not always being comparable or reproducible. The use of standardized procedures for sample storage as well as the use of samples stored in biobanks should be promoted to obtain high-quality samples for biomarker discovery.

## Supplementary Information

**Additional file 1.** Preferred Reporting Items for Systematic Reviews and Meta-Analyses (PRISMA) standards and guidelines.

**Additional file 2.** New Castle Ottawa Scale (NOS) for case-control study quality assessment.

## Abbreviations

ASCO: American Society of Clinical Oncology
BC: Breast cancer
CA 15-3: Cancer antigen 15-3
CLEIA: Chemiluminescent enzyme immunoassay
CMIA: Chemiluminescent microparticle immunoassay
EGTM: European Group on Tumor Markers
EIA: Enzyme immunoassay
ELISA: Enzyme-linked immunosorbent assay
ESMO: European Society for Medical Oncology
MEIA: Microparticle enzyme immunoassay
NACB: National Academy of Clinical Biochemistry
NCCN: National Comprehensive Cancer Network
NOS: Newcastle-Ottawa scale
PRISMA: Preferred Reporting Items for Systematic Reviews and Meta-Analyses
QC: Quality control
RIA: Radioimmunoassay
