# Supplementary material for: The impact of different preanalytical methods related to CA 15-3 determination in frozen human blood samples: a systematic review
Source: Syst Rev. 2021 Apr 9;10:102. doi: 10.1186/s13643-021-01631-7 (PMC8033739; doi:10.1186/s13643-021-01631-7)
Supplement: Supplementary file 2 — Additional file 2. New Castle Ottawa Scale (NOS) for case-control study quality assessment. [file 13643_2021_1631_MOESM2_ESM.docx]

**Additional file 2:** New Castle Ottawa Scale (NOS) for case-control study quality assessment.

|  | ***NOS. SELECTION*** | | | | ***NOS. COMPARABILITY*** | ***NOS. EXPOSURE*** | | | **NOS SCORE** |
| --- | --- | --- | --- | --- | --- | --- | --- | --- | --- |
| **STUDY/YEAR** | **CASE SELECTION** | **REPRESENTATIVE CASES** | **CONTROL SELECTION** | **DEFINITION OF CONTROLS** | **COMPARABILITY OF CASES AND CONTROLS** | **ASCERTAINMENT OF EXPOSURE** | **ASCERTAINMENT SOME METHODS** | **NON-RESPONSE RATE** |  |
| Chukwurah EF et al. 2018 | **c** | **a1** | **a1** | **b** | **a1 b0** | **d** | **a1** | **c** | **4** |
| Mahmood M. et al 2016 | **b** | **a1** | **a1** | **b** | **a1 b0** | **d** | **a1** | **c** | **4** |
| Said NM et al 2019 | **b** | **a1** | **b** | **a1** | **a1 b0** | **c** | **a1** | **c** | **4** |
| Moazzezy N et al 2014 | **b** | **a1** | **c** | **b** | **a1 b1** | **c** | **a1** | **c** | **4** |
| El-Moneim Ebied S A et al 2013 | **b** | **b** | **c** | **a1** | **a1 b1** | **d** | **a1** | **c** | **4** |
| Svobodova S et al. 2018 | **b** | **b** | **c** | **b** | **a1 b0** | **d** | **a1** | **c** | **2** |
| Pedersen AC et al 2013 | **b** | **b** | **c** | **b** | **a1 b0** | **e** | **a1** | **c** | **2** |
| Di Gioia D et al. 2015 | **c** | **b** | **a1** | **a1** | **a1 b0** | **d** | **a1** | **c** | **4** |
| Sen S et al. 2011 | **a1** | **a1** | **c** | **b** | **a1 b1** | **e** | **a1** | **c** | **5** |
| Metwally FM et al. 2010 | **b** | **a1** | **b** | **b** | **a1 b0** | **a1** | **a1** | **c** | **4** |
| Hewala TI et al. 2012 | **b** | **a1** | **c** | **b** | **a1 b1** | **d** | **a1** | **c** | **4** |
| Zajkowska M et al. 2020 | **b** | **b** | **a1** | **b** | **a1 b0** | **d** | **a1** | **c** | **3** |
| Zaleski M et al. 2018 | **b** | **a1** | **b** | **a1** | **a1 b0** | **d** | **a1** | **d** | **4** |
| Tang Z et al. 2018 | **c** | **a1** | **c** | **c** | **a0 b1** | **e** | **a1** | **c** | **3** |
| Christenson RH et al. 2011 | **c** | **b** | **c** | **a1** | **a1 b0** | **d** | **a1** | **c** | **3** |
| Saba MA et al. 2017 | **b** | **a1** | **b** | **b** | **a1 b1** | **d** | **a1** | **c** | **4** |
| Laidi F et al. 2014 | **c** | **a1** | **a1** | **a1** | **a1 b1** | **c** | **a1** | **c** | **6** |
| Ercan Ş et al. 2012 | **b** | **a1** | **b** | **a1** | **a1 b1** | **e** | **a1** | **b** | **5** |

**NEWCASTLE - OTTAWA QUALITY ASSESSMENT SCALE CASE CONTROL STUDIES – CODING MANUAL**

Note: A study can be awarded a maximum of one star for each numbered item within the Selection and Exposure categories. A maximum of two stars can be given for Comparability.

**Selection**

1) Is the case definition adequate?

a) yes, with independent validation ****

b) yes, eg record linkage or based on self reports

c) no description

2) Representativeness of the cases

a) consecutive or obviously representative series of cases ****

b) potential for selection biases or not stated

3) Selection of Controls

a) community controls ****

b) hospital controls

c) no description

4) Definition of Controls

a) no history of disease (endpoint) ****

b) no description of source

**Comparability**

1) Comparability of cases and controls on the basis of the design or analysis

a) study controls for **SAMPLE FREEZE-THAW CYCLE AND HANDLING** (Select the most important factor.) ****

b) study controls for any additional factor **BALANCED SAMPLE SIZE** (This criteria could be modified to indicate specific control for a second important factor.)

**Exposure**

1) Ascertainment of exposure

a) secure record (eg surgical records) ****

b) structured interview where blind to case/control status ****

c) interview not blinded to case/control status

d) written self report or medical record only

e) no description

2) Same method of ascertainment for cases and controls

a) yes ****

b) no

3) Non-Response rate

a) same rate for both groups ****

b) non respondents described

c) rate different and no designation
